# Glutathione-S-transferases in lung and sputum specimens, effects of smoking and COPD severity

**DOI:** 10.1186/1465-9921-9-80

**Published:** 2008-12-13

**Authors:** Terttu Harju, Witold Mazur, Heta Merikallio, Ylermi Soini, Vuokko L Kinnula

**Affiliations:** 1 Institute of Clinical Medicine, Department of Internal Medicine, Centre of Excellence in Research, P. O. Box 5000, 90014 University of Oulu, Oulu, Finland; 2 Department of Internal Medicine, Clinical Research Center, Oulu University Hospital, Oulu, Finland; 3 Department of Medicine, Division of Pulmonary Diseases, University of Helsinki and Helsinki University Hospital, Helsinki, Finland; 4 Department of Pathology, Oulu University Hospital, Oulu, Finland; 5 Department of Clinical Pathology and Forensic Medicine, University of Kuopio, Kuopio, Finland

## Abstract

**Background:**

Oxidative stress plays a potential role in the pathogenesis and progression of chronic obstructive pulmonary disease (COPD). Glutathione S-transferases (GSTs) detoxify toxic compounds in tobacco smoke via glutathione-dependent mechanisms. Little is known about the regulation and expression of GSTs in COPD lung and their presence in airway secretions.

**Methods:**

GST alpha, pi and mu were investigated by immunohistochemistry in 72 lung tissue specimens and by Western analysis in total lung homogenates and induced sputum supernatants from non-smokers, smokers and patients with variable stages of COPD severity.

**Results:**

GST alpha was expressed mainly in the airway epithelium. The percentage of GST alpha positive epithelial cells was lower in the central airways of patients with very severe (Stage IV) COPD compared to mild/moderate COPD (p = 0.02). GST alpha by Western analysis was higher in the total lung homogenates in mild/moderate COPD compared to cases of very severe disease (p < 0.001). GST pi was present in airway and alveolar epithelium as well as in alveolar macrophages. GST mu was expressed mainly in the epithelium. Both GST alpha and pi were detectable in sputum supernatants especially in patients with COPD.

**Conclusion:**

This study indicates the presence of GST alpha and pi especially in the epithelium and sputum supernatants in mild/moderate COPD and low expression of GST alpha in the epithelium in cases of very severe COPD. The presence of GSTs in the airway secretions points to their potential protective role both as intracellular and extracellular mediators in human lung.

## Background

Epithelial lining fluid (ELF) contains more than 140-fold higher levels of glutathione (GSH) (L-γ-glutamyl-L-cysteinyl-glycine) compared to plasma, evidence of its critical role in protecting airway epithelium from oxidant injury [1,2]. Glutathione S-transferases (GSTs) consist of a superfamily of dimeric phase II metabolic enzymes that catalyze the conjugation of reduced GSH with electrophilic compounds e.g. detoxifing toxic components of tobacco smoke. They are mainly regulated by nuclear factor, erythroid-derived 2, like 2 (Nrf2), a transcription factor that contributes to the induction of several protective enzymes during oxidative stress [3-5]. In experimental animals exposed to cigarette smoke, inhibition of this system in turn leads to emphysema [6,7], another indicator of the importance of this mechanism and related enzymes such as GSTs in the prevention of chronic obstructive pulmonary disease (COPD)/emphysema. The protein levels of several GSTs with the exception of GST omega [8] have not been investigated in human COPD. Furthermore, little is known about their possible presence in airway secretions of healthy or COPD lung. Given the high levels of GSH in the epithelial lining fluid, intracellular vs extracellular GSH homeostasis may be partly regulated by the GSTs, enzymes that participate both in GSH transport and detoxification reactions.

There are a number of GST isoenzymes including GST alpha (GSTA), mu (GSTM), pi (GSTP), omega, theta, sigma, and kappa. In proximal airways, GST pi, mu and alpha have been found in the brush border and GST pi and mu but not GST alpha in alveolar cells and macrophages [9] while other GSTs have not been investigated in the peripheral lung. The RNA levels of GSTA2 and GSTP1 are elevated in the bronchial epithelium of smokers but no such difference has been found for GSTM1 [10,11]. As far as we are aware there is only one microarray study on the RNA levels of antioxidant enzymes including GSTs in the bronchial brushings of COPD patients, which indicated that though the RNA expression of these enzymes may change, there does not seem to be a linear correlation with COPD severity [12].

Genetic polymorphisms of xenobiotic metabolizing enzymes including GSTs have been shown to associate with COPD in many previous investigations. GSTP1 gene polymorphism correlates with susceptibility to COPD [13] and homozygous deletion of the GSTM1 gene is associated with emphysema in patients who have lung cancer [14] and with chronic bronchitis in heavy cigarette smokers [15]. Polymorphism of GSTO2, is associated with low lung function values [16]. The GSTM1, GSTT1 null, and GSTP1 Val/Val have been linked with increased risk (12-fold) for COPD [17] and GSTT1 deficiency in combination with GSTM1 deficiency independently appears to be associated with an accelerated age-related decline of lung function in males irrespective of smoking [18]. Most of these studies on GST polymorphisms highlight the importance of these enzymes in protection against the oxidative stress induced by cigarette smoke.

This study was undertaken 1) to investigate the distribution and expression of GSTs in normal human lung and COPD of various severities both in proximal airways and in peripheral lung tissue and 2) to study the expression of the GSTs in induced sputum cells and supernatants in healthy individuals and patients with COPD.

## Materials and methods

### Lung tissue specimens

Lung tissue specimens from 72 patients (16 life-long non-smokers, 26 current smokers with mild-to-moderate COPD, 22 current smokers with normal lung function) operated on for local lung tumour (malignant and non-malignant such as hamartomas) and 8 ex-smokers with very severe (Stage IV) COPD undergoing lung transplantation formed the basis for immunohistochemical studies from Oulu and Helsinki University Hospitals. Tissue specimens from tumor-free resection line and from the peripheral lung tissue were selected. COPD was defined on the basis of preoperative lung function: FEV1/FVC less than 70% and no reversibility (bronchodilatation effect less than 12%). The patients were not receiving corticosteroid therapy (neither inhaled nor systemic) with the exception of the lung transplantation cases. The clinical characteristics were obtained from the patient records (Table 1).

**Table 1:** Patient characteristics of the specimens examined by immunohistochemistry

	Non-smoker N = 16	Smoker N = 22	COPD N = 34	p-value
Age, years	65 (13)	63 (8)	62 (9)	0.543
Sex M:F	8:8	17:5	27:7	0.103
Pack-years	0	46 (19)*	38 (13)§	0.000
FEV1 %predicted	98 (15)	90 (10)	55 (23)*#	0.000
FEV1/FVC %	86 (9)	83 (11)	56 (15)*#	0.000
MEF50 %pred	94 (24)	80 (37)	34 (21)*#	0.000
DCO %pred	91 (15)	78 (14)	64 (27)§	0.004
DCO/VA %pred	89 (11)	83 (12)	72 (24) §	0.035

Mean (SD)

*The mean difference between non-smokers and smokers is significant at the 0.05 level, Dunnett t-test.

§The mean difference between non-smokers and COPD-patients is significant at the 0.05 level, Dunnett t-test.

# The mean difference between smokers and COPD-patients is significant at the 0.05 level, Dunnett t-test.

Lung tissue specimens from peripheral lung tissue for the Western analyses had been frozen immediately after the surgery in liquid nitrogen. They were homogenized in ice cold phosphate buffered saline (PBS), used for Western analysis and evaluated for the total level of GSTs alpha, mu and pi in the lung. (Table 2).

**Table 2:** Patient characteristics in the specimens examined by Western analysis.

	Non-smoker N = 7	Smoker N = 7	COPD N = 16	p-value
Age, years	63 (13)	59 (4)	62 (10)	0.754
Sex M:F	4:3	5:2	12:4	0.688
Pack-years	0	36 (16)*	34 (17)§	0.000
FEV1 %predicted	88 (18)	88 (13)	55 (31)*#	0.005
FEV1/FVC %	83 (9)	83 (10)	57 (20)*#	0.000
MEF50 %pred	88 (35)	84 (61)	37 (22)	0.066
DCO %pred	95 (20)	82 (10)	52 (22)§	0.003
DCO/VA %pred	100 (18)	89 (9)	62 (24) §	0.006

Mean (SD)

All cases were studied for the expression of GST pi, but due to the exhaustion of biopsy material, only 4 non-smokers, 3 smokers and 8 COPD-cases were included in the Western analysis for GST alpha expression and 4 smokers, 4 non-smokers and 10 COPD-patients for GST mu expression.

*The mean difference between non-smokers and smokers is significant at the 0.05 level, Dunnett t-test.

§ The mean difference between non-smokers and COPD-patients is significant at the 0.05 level, Dunnett t-test.

# The mean difference between smokers and COPD-patients is significant at the 0.05 level, Dunnett t-test.

### Induced sputum

Induced sputum samples from 3 healthy non-smokers, 3 symptomatic smokers (chronic bronchitis) and 6 smokers with COPD formed the material for sputum experiments. Sputum was induced by inhalation of 4.5% hypertonic saline given at 5-minute intervals for a maximum of 20 minutes according to the guidelines of the European Respiratory Society's Task Force [19]. The characteristics of the patients selected for the studies on induced sputum specimens are shown in Table 3.

**Table 3:** The characteristics of the patients providing sputum samples

	Non-smoker N = 3	Chronic bronchitis N = 3	COPD N = 6	p-value
Age, years	45 (21)	44 (16)	60 (7)	0.181
Sex M:F	3:0	3:0	4:2	0.301
Pack-years	0	29 (15)	48 (18)	0.005
FEV1 %predicted	113 (19)	101 (4)	61 (21)	0.021
FEV1/FVC %	88 (2)	80 (1)	64 (15)	0.115
MEF50 %pred	131 (47)	87 (12)	36 (20)	0.006
DCO %pred	92 (7)	95 (6)	63 (24)	0.053
DCO/VA %pred	100 (5)	99 (8)	75 (31)	0.266

Mean (SD)

### Immunohistochemistry

The sections were deparaffinized in xylene and rehydrated in a descending ethanol series. Endogenous peroxidase was blocked by incubating the sections in 3% hydrogen peroxide in absolute methanol for 15 minutes. The sections were incubated with the primary polyclonal antirabbit antibodies for GST mu (1:100), GST pi (1:100) or GST alpha 1:75 (Acris Antibodies, Hiddenhausen, Germany). The immunostaining was done using the Histostain-Plus Kit (Zymed Laboratories Inc., San Francisco, CA), and the chromogen was aminoethyl carbazole (AEC) (Zymed Laboratories Inc.). In negative controls, the primary antibody was substituted with phosphate-buffered saline (PBS) or rabbit primary antibody isotype control from Zymed Laboratories Inc.

For GST alpha, GST mu and GST pi, the immunoreactivity was assessed semiquantitatively from all fields of one section of central and peripheral lung by separately grading the staining intensity of the macrophages, bronchial, bronchiolar or alveolar epithelium or vascular endothelium as negative (0), weak (1) or moderate (2) or intense (3) (YS). For GST alpha, the immunoreactivity was concentrated in the airway epithelium and the grading of GST alpha immunoreactivity was quantified by estimating the percent of positive epithelial cells (YS).

Immunocytochemistry of GST alpha in induced sputum cells was performed as previously described by Peltoniemi M et al [20]. The cytospin samples were treated with Ortho Permeafix (Ortho Diagnostic Systems Inc., UK) and for immunostaining, Zymed ABC Histostain-Plus Kit was used according to the manufacturer's protocol. The samples were incubated with an antibody against GST alpha and negative control samples with Zymed Rabbit Isotype Control and PBS, and stained with AEC (Zymed Laboratories Inc.) and thereafter with Mayer's haematoxylin.

### Western analysis

The cell pellets were resuspended in sterile water containing protease inhibitors, Complete Mini tablets (Roche, Mannheim, Germany) and cells were lysed by sonicating. The protein concentrations were measured using the DC protein assay from Bio-Rad (Bio-Rad Laboratories, Hercules, CA, USA) and 40 μg of cell protein was applied per lane to a 12% sodium dodecyl sulphate-polyacrylamide gel (SDS-PAGE) and electrophoresed as described [20]. Due to the major changes in the classical constitutive proteins that have been generally used as loading controls [21-23], here the equal loading was ensured by Ponceau protein assay after careful protein determinations. Membranes were incubated with primary antibodies against GST alpha, mu and pi (dilution 1:4000 for GST alpha, 1:2000 for GST pi and 1:1000 for GST mu) for 3 hours followed by treatment with secondary antibodies over night in +4°C. Protein bands were detected with Immobilon detection solution (Millipore, Billerica, MA, USA) and the luminal excitation was imaged on *x *ray film (Amersham Biosciences, Buckinghamshire, UK). Quantification of the band sum intensity was used the Kodak 1D Scintific Image Analysis System (Eastman Kodak Company, New Haven, Connecticut, USA).

### Statistical methods

The statistical analyses were performed with the SPSS for Windows software (SPSS, Chicago, IL, USA). Continuous data were compared using analysis of variance (ANOVA). When ANOVA results indicated that groups differed, post hoc comparisons were performed using two-tailed t-tests.

Categorical data were compared using Fisher exact test designed for small sample groups. P-values less than 0.05 were considered statistically significant.

### Ethical considerations

The study protocol was accepted by the ethical committee of the University of Oulu and Oulu University Hospital and the ethical board of Helsinki University Hospital and it is in accordance with the ethical standards of the Helsinki declaration of 1975.

## Results

### GST alpha

**Figure 1:**
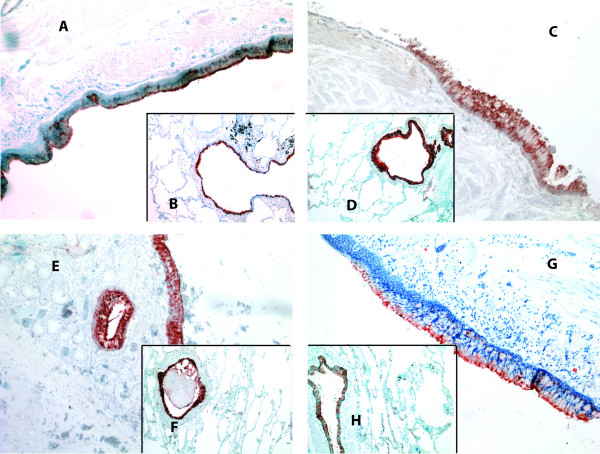
**Immunohistochemical staining for GST alpha in specimens from the central and peripheral lung of a non-smoker (A and B, respectively), a smoker (C and D), and patients with Stage I-II COPD (E and F) and Stage IV COPD (G and H)**. GST alpha was mainly located in the airway epithelium.

**Figure 2:**
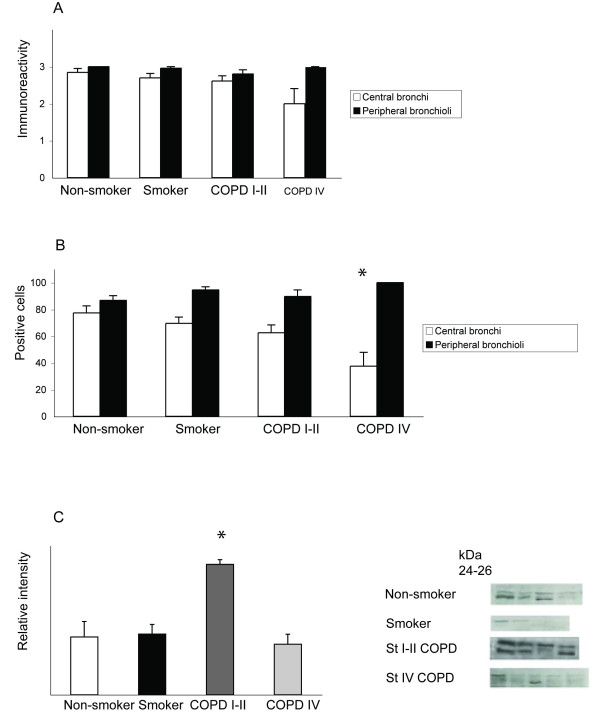
**GST alpha expression in the airways and in total lung homogenates**. A. The GST alpha immunoreactivity in airway epithelium of central and peripheral airways. The staining was graded as negative (0), weak (1) or moderate (2) or intense (3). The GST alpha immunoreactivity was strong in the epithelium of both large, cartilaginous airways as well as in the epithelium of small peripheral bronchioli. There was a trend for diminished immunoreactivity in cases of very severe (Stage IV) COPD but the difference between the groups was not statistically significant. The means are shown as columns with error bars representing SEM. B. The percentage of GST alpha positive epithelial cells was observed to decrease in the large airways of the patients with very severe (Stage IV) COPD compared to non-smokers (p = 0.02). C. Western analysis of GST alpha in the lung homogenates of healthy non-smokers and smokers and in patients with different stages of COPD showed an increased immunoreactivity in patients with Stage I-II COPD compared to non-smokers or smokers (p = 0.007). The means of the measured sum intensities are shown as columns with error bars representing SEM.

**Figure 3:**
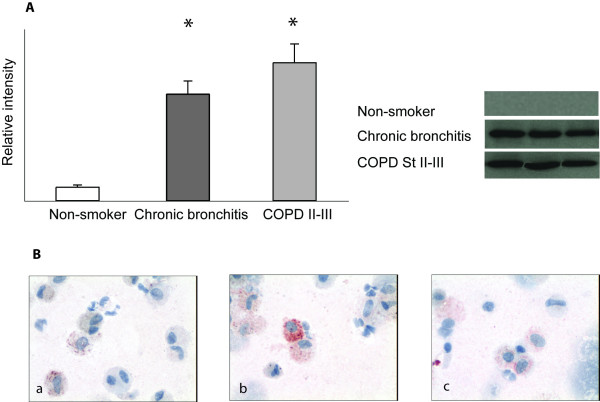
**GST alpha immunoreactivity in sputum cells and supernatants**. A. Western analysis for GST alpha in induced sputum supernatants revealed an increased immunoreactivity in patients with chronic bronchitis and in Stage II-III COPD compared to healthy non-smokers (p < 0.001). The means of the measured intensities are shown as columns with error bars representing SEM. B. Representative sputum cytospins from a smoker (a), patient with chronic bronchitis (b) and patient with Stage II COPD (c). Macrophages in the induced sputum exhibited positive GST alpha reactivity.

GST alpha was mainly localized in the central and peripheral airway epithelium, with some alveolar macrophages showing weak positivity (Figure 1). The intensity of GST alpha staining showed a tendency to be lower in the central airways of cases of very severe (Stage IV) COPD compared to Stage I-II COPD. Moreover, the percentage of GST alpha positive epithelial cells was significantly lower in the central airway epithelium of Stage IV COPD than in Stage I-II COPD (p = 0.02). No corresponding changes could be seen in the peripheral airway epithelium (Figure 2A, B). When the total immunoreactivity was assessed by Western analysis of the lung homogenate, GST alpha was higher in Stage I-II COPD (p < 0.001) than in Stage IV COPD (Figure 2C). Additionally, GST alpha was clearly detectable in induced sputum supernatants, being higher both in chronic bronchitis and in Stage II-III COPD than in the healthy non-smokers (Figure 3) (p < 0.001). The Western blotting of GST alpha consistently showed two bands from the tissues but not from sputum supernatants. These results can be related to many reasons one of those being proteolysis of GST alpha in the tissues. Another, even more likely reason is the presence of various alpha-class GSTs in human lung tissues but not in sputum supernatants. Two GST alpha subtypes have been earlier documented in rat tissue homogenates and porcine Sertoli cells and our results are in full agreement with those investigations [24,25]. In induced sputum cytospins, GST alpha was localized in macrophages (Figure 3). Induced sputum was not collected from very severe cases partly due to technical difficulties, only lung tissue specimens were available from the cases of very severe COPD.

### GST pi

**Figure 4:**
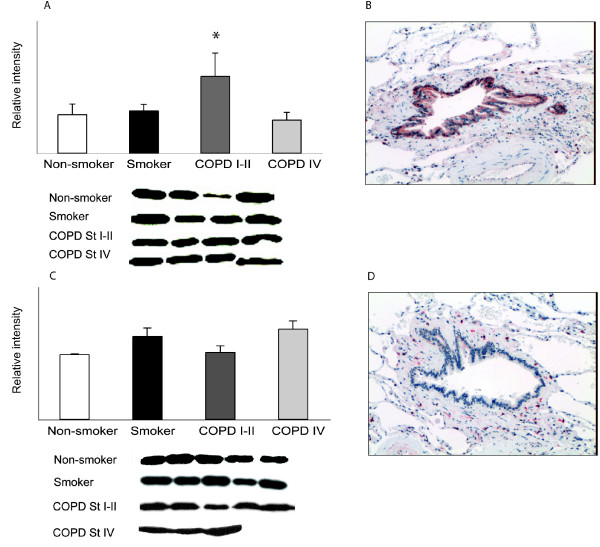
**GST pi and mu immunoreactivities in human lung**. A. In Western analysis of GST pi in lung homogenates, the level of GST pi was elevated in Stage I-II COPD compared to that in healthy smokers (p = 0.002). B. In immunohistochemical staining, GST pi was mainly expressed in the epithelium of airways and alveoli. C. Western analysis of the lung homogenates for GST mu showed some variability but the difference between the groups was not significant (p = 0.063). C. In immunohistochemical staining, GST mu was located in the macrophages; the expression in bronchial and alveolar epithelium was weak.

GST pi was present in the epithelium of airways and alveoli and in approximately 5% of alveolar macrophages. The immunoreactivity or the number of GST pi positive cells did not differ between the various COPD severities. When lung tissue homogenates were evaluated by Western analysis, GST pi was higher in Stage I-II COPD compared to healthy smokers (p = 0.002) (Figure 4A,B). GST pi could also be detected from the sputum supernatants but there were no significant differences between controls and COPD patients.

### GST mu

GST mu was located in the macrophages, bronchial and alveolar epithelium. Western analysis of the lung homogenates for GST mu exhibited some variability (Figure 4C,D) but the changes were minor and not significant (p = 0.063). Sputum supernatant was negative for GST mu in the Western blot analyses, but this negative finding does not exclude its presence in airway secretions.

## Discussion

The results of the present investigation extend previous knowledge about GSTs and human lung which so far have mainly focused on the polymorphisms in these enzymes in protecting human lung against toxic metabolites [26] or the mRNA levels of these enzymes in the bronchial brushings of smokers or COPD patients [11,12]. Here the actual GST proteins were expressed in large and peripheral airways, where the levels of GST alpha were low in the large airways of cases with very severe (Stage IV) COPD. We present another new finding which may have important role in the interpretation of the antioxidant defense of airway epithelial lining fluid in general, i.e. the presence GST alpha in particular in sputum supernatants and its further elevation in chronic bronchitis and COPD. There was clear immunoreactivity of GST alpha in sputum macrophages but the staining positivity cannot exclude the possibility that macrophages may also have ingested GST alpha positive material from the epithelial lining fluid/sputum supernatant. In patients with very severe disease, the decline of GST activity can significantly worsen the imbalance between oxidants and GSH associated detoxification mechanisms in the airways.

Our findings are in line with the results of the microarray analysis performed by Hackett et al. describing upregulation of GST A2 RNA in airway epithelium of smokers [11]. In that particular study, no changes in GST M3, M4 or GST pi RNA expression could be observed. In a recent study, also GSTM3 gene expression was upregulated in healthy smokers and in COPD compared to healthy smokers [12]. However, the RNA level did not increase linearly as the disease progressed. It is known that the RNA level does not necessarily correlate with the protein or functional activity. In the study of Pierrou and co-workers for example, levels of CYP1B1 RNA but not the immunoreactive protein were elevated in the bronchial brushings of COPD patients. The present study on several GSTs revealed the most marked immunoreactivity and greatest changes in GST alpha in COPD. The strength of our study is the large material including non-smokers, smokers and patients in different stages of COPD, with specimens from central and peripheral airways, pointing to the importance of GSTs, especially GST alpha against smoking induced oxidative stress. This study confirms our previous findings, that the protein levels of many antioxidant enzymes which are involved in GSH homeostasis of the lung do not increase linearly as the disease progresses and may even be downregulated as the disease progresses to terminal stages [8,20,27]. Whether the immunoreactivity of a specific GST correlates with the corresponding isoenzyme activity remains unclear. The COPD Stage IV patients were ex-smokers whereas Stage I-II COPD cases were current smokers. Further studies will be needed to evaluate the effects of smoking cessation in this and other protective enzymes in human lung.

The levels of GST alpha decreased in very severe COPD. This is in agreement with earlier studies with GST omega [8], glutaredoxin [20] and the rate limiting enzyme in the synthesis of GSH i.e. glutamate-cysteine ligase [27]. All these enzymes are regulated by a Nrf2 related mechanism, where Nrf2, Keap1 and the Nrf2 stabilizer DJ-1 are involved. Importantly, a recent study on these mechanisms in COPD revealed a decline in Nrf2 protein and mRNA levels and decreased DJ-1 levels in cases of severe COPD [28].

The maintenance of GSH in the epithelial lining fluid (ELF) and its elevation in the ELF of smokers [29-31] are poorly understood. GSH is transported from the cells by multiple mechanisms including GST dependent detoxification reactions. In the extracellular milieu, GSH can be bound to proteins and/or be further degraded to amino acids which then move inside the cells to participate in GSH re-synthesis. The toxins present in the cigarette smoke can be transported from the cells by GSTs in reactions that consume GSH. The expression of GST alpha and pi in induced sputum supernatants suggests that after initial induction which occurs via a Nrf2 mediated mechanism, GSTs, at least GST alpha, pi and omega [8] can be exported/secreted to the extracellular space. The GSTs in the sputum supernatant fractions observed in this study and in earlier studies are not artifacts related to cell disruption during the isolation, since other intracellular markers for cell lysis were negative [20]. GSTs, in combination with GSH which can also be released from the proteins by glutaredoxin, can further detoxify a number of reactive compounds in the extracellular milieu. This initial increase followed by the subsequent decline of several enzymes related to GSH synthesis/homeostasis including GST in COPD as shown in this study and in the previous investigations parallels with the initial induction and consequent dysfunction of the Nrf2 pathway in COPD lungs.

To conclude, this study on GSTs shows the presence of GST alpha especially in mild/moderate COPD. These results combined with previous studies on the major antioxidant enzymes suggest early induction but consequent decline of antioxidant defense systems related to the Nrf2 pathway in severe/very severe COPD. This study remarkably extends earlier observations since this is also the first one in detecting elevated GST alpha levels in the sputum supernatants in chronic bronchitis and in COPD a situation with documented increased oxidant burden and inflammation of the airways.

## Competing interests

The authors declare that they have no competing interests. The study has not been supported by tobacco industry.

## Authors' contributions

TH participated in the design of the study and selection of patient material, performed part of the statistical analysis and drafted the manuscript. WM participated in selection and collection of patient material, analyzing the Western analysis results and performed part of the statistical analysis and participated in creating the figures. HM carried out the Western analyses and participated in creating the figures. YS participated in selection of patient material and analyzed the immunohistochemical results. VLK conceived the study, and participated in its design and coordination and helped to draft the manuscript. All authors have read and approved the final manuscript.
